# Supplementary material for: Observation of non-reciprocal harmonic conversion in real sounds
Source: Commun Phys. 2023 May 6;6(1):93. doi: 10.1038/s42005-023-01217-w (PMC11041789; doi:10.1038/s42005-023-01217-w)
Supplement: Supplementary file 2 — Suplementary information [file 42005_2023_1217_MOESM2_ESM.pdf]

Supplementary information for:

Observation of non-reciprocal harmonic conversion in real sounds

Xinxin Guo,[1] Hervé Lissek,[1] and Romain Fleury[2,a)]

[1)] *Signal Processing Laboratory LTS2, Ecole Polytechnique Fédérale de Lausanne, 1015 Lausanne, Switzerland*

[2)] *Laboratory of Wave Engineering, Ecole Polytechnique Fédérale de Lausanne, 1015 Lausanne, Switzerland*

[a)] Corresponding author. Email: romain.fleury@epfl.ch

SUPPLEMENTARY NOTE 1: EXPERIMENTAL SETUP

The unit cells used are made of commercially-available loudspeakers Visaton FRWS 5, each with 3D-printed enclosures, as shown in Supplementary Figure. 1a. The Thiele/Small parameters of each closed-box resonator are given in the table of Supplementary Figure. 1b. They are derived experimentally based on two calibration measurements of the acoustic impedance, i.e., the first in the open circuit case and the second in the short circuit case. A photograph of the setup is given in Supplementary Figure. 1c where we employ an anechoic termination to ensure an absorption coefficient higher than 0.998 for the whole frequency range of interest (from 180 Hz). The 4 microphones on the waveguide, mic 1 to mic 4 in Supplementary Figure. 1c, are used for the measurements of transmission coefficient (in the linear cases). They are placed with a distance of 5 cm between mic 1 and mic 2 (also between mic 3 and mic 4), and a distance of 45 cm between mic 2 (mic 3) and the center of the liner. The pressure sensed by the microphone closest to the anechoic end is recognized as the final transmitted wave.

a

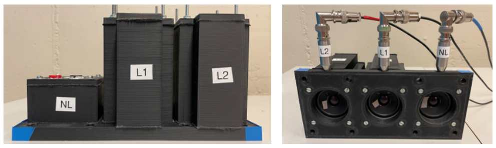

b

Cell	M_{ms}(g)	R_{ms}(N.s.m^{-1})	C_{mc}(mm.N^{-1})	Bl(N.A^{-1})	f_0(Hz)
'NL'	0.54	0.27	0.27	1.34	449
'L1'	0.55	0.26	0.24	1.38	439
'L2'	0.55	0.27	0.14	1.43	580

c

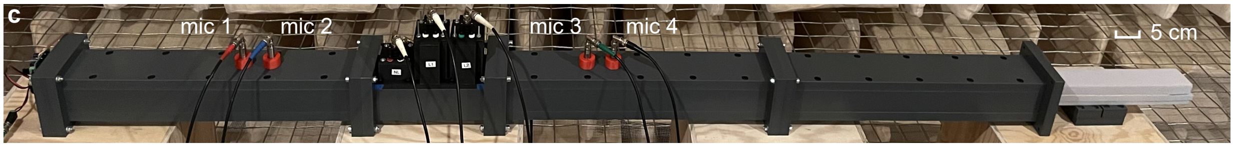

Supplementary Figure 1. **Experimental setup. a** The three unit cells in use, labeled by NL, L1 and L2, respectively, each with one microphone in front for applying the specified active control. **b** The Thiele/Small parameters of the unit cells. **c** The setup employed in measurements containing an anechoic termination.

SUPPLEMENTARY NOTE 2: LINEAR AND NONLINEAR CONTROLS

We consider the impedance control scheme developed by E. Rivet[1] to perform the linear controls on the unit cells L1 and L2, as schematically presented in Supplementary Figure. 2a. For a closed-box loudspeaker and in the passive open circuit case, the specific acoustic

impedance of the diaphragm $Z_{\mathrm{as}}(\mathrm{j}\omega)$ can be obtained in the frequency domain as

$$Z_{\mathrm{as}}(\mathrm{j}\omega) = \frac{P_{\mathrm{f}}(\mathrm{j}\omega)}{V(\mathrm{j}\omega)} = \mathrm{j}\omega\frac{M_{\mathrm{ms}}}{S_{\mathrm{d}}} + \frac{R_{\mathrm{ms}}}{S_{\mathrm{d}}} + \frac{1}{\mathrm{j}\omega C_{\mathrm{mc}} S_{\mathrm{d}}}, \tag{1}$$

where the uppercase symbols P_{f} and V are used to represent the frequency responses of the considered acoustic quantities.

The considered impedance control refers to the following control law with a transfer function Φ:

$$\Phi(\mathrm{j}\omega) = \frac{I(\mathrm{j}\omega)}{P_{\mathrm{f}}(\mathrm{j}\omega)} = \frac{S_{\mathrm{d}}}{B\ell}\frac{Z_{\mathrm{st}}(\mathrm{j}\omega) - Z_{\mathrm{as}}(\mathrm{j}\omega)}{Z_{\mathrm{st}}(\mathrm{j}\omega)}. \tag{2}$$

It can be easily deduced that with the such expression of feedback current, the specific acoustic impedance of the controlled resonator, namely the ratio between pressure source and velocity of the diaphragm, can be changed from the natural impedance $Z_{\mathrm{as}}(\mathrm{j}\omega)$ into a target one $Z_{\mathrm{st}}(\mathrm{j}\omega)$.

A form similar to the passive case is considered to define the target acoustic impedance:

$$Z_{\mathrm{st}}(\mathrm{j}\omega) = \mathrm{j}\omega\mu_{\mathrm{M}}\frac{M_{\mathrm{ms}}}{S_{\mathrm{d}}} + \mu_{\mathrm{R}}\frac{R_{\mathrm{ms}}}{S_{\mathrm{d}}} + \frac{\mu_{\mathrm{C}}}{\mathrm{j}\omega C_{\mathrm{mc}} S_{\mathrm{d}}}, \tag{3}$$

where μ_{M}, μ_{C} and μ_{R} represent three control parameters to adjust the moving mass M_{ms}, the compliance C_{mc} and the resistance R_{ms} of the resonator, respectively.

The resonance frequency is prescribed by tuning the ratio $\mu_{\mathrm{C}}/\mu_{\mathrm{M}}$. The resonance magnitude is altered by the control parameter μ_{R} which we define always to be 0.05 for performing the desired resistance minimization. The effective bandwidth of resonance is either fixed by setting $\mu_{\mathrm{M}} = 1$ for pure tones or is enlarged with $\mu_{\mathrm{M}} = 0.5$ to handle the wave-packet excitation and the musical instrument notes. The transmission blocking realized through the presented linear impedance controls is showcased in Supplementary Figure. 2b, for three target frequencies $f_1 = 190\,\mathrm{Hz}$, $f_2 = 265\,\mathrm{Hz}$ and $f_3 = 400\,\mathrm{Hz}$, respectively.

As for the non-linear control applied to the unit cell NL, a simple quadratic control law is implemented as explained in the main manuscript. Supplementary Figure. 2c shows the evolution of the first six harmonic components ($n\omega$ with $n = 1, 2, ..., 6$) extracted from the transmitted wave when only non-linear control is activated. Pure tone excitation at the same frequencies f_i ($i = 1, 2, 3$) is considered in Supplementary Figure. 2b. As the nonlinear control gain G_{NL} increases, the second harmonic 2ω is always the first to manifest due to the control-provided quadratic nonlinearity. It is further enhanced when the generated 2ω is close to the resonance frequency of the controlled resonator, as we would expect and as

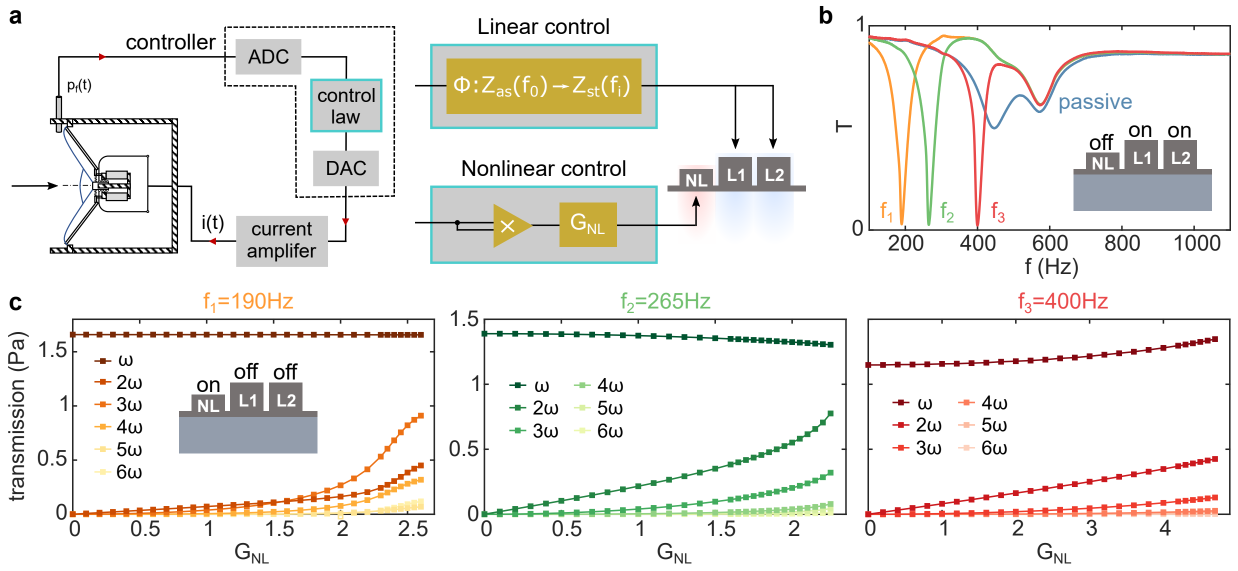

Supplementary Figure 2. **The effects of linear and non-linear controls.** **a** The control strategies: applying non-linear quadratic control on the unit cell indicated by NL, and linear impedance controls on the other two with labels of L1 and L2 respectively. The Analog-to-Digital and the Digital-to-Analog Converters (ADC and DAC) contained in the control system allow for more complex control laws to be implemented directly in a digital way. **b** The transmission coefficient T of the system measured when only linear controls are turned on, targeting different frequencies f_i $(i = 1, 2, 3)$. **c** The generation of higher harmonics (the first six harmonics) in the transmitted wave when only non-linear control is switched on, as the gain parameter G_{NL} defined in **a** increases and under pure tone excitation of the same frequencies as aimed in **b**.

demonstrated by the case of $f_2 = 265\,\mathrm{Hz}$ in Supplementary Figure. 2c. Meanwhile, other higher harmonics also increase sequentially as the nonlinearity strengthens, while affecting the fundamental component (at ω) through interactions between adjacent harmonics (as the cases of $f_3 = 400\,\mathrm{H}z$ and $f_2 = 265\,\mathrm{Hz}$). If a higher harmonic such as the third one 3ω lies around the resonance of the controlled unit, it can then stand out and even surpass the second harmonic, as revealed in the case of $f_1 = 190\,\mathrm{Hz}$. As expected, in the case where all higher harmonics are far from the resonance, such as the case of $f_3 = 400\,\mathrm{H}z$, their generations are not very efficient, as can be noticed by comparing the results of $f_3 = 400\,\mathrm{H}z$ and $f_2 = 265\,\mathrm{Hz}$ in Supplementary Figure. 2c. However, we prove in the main manuscript that such inefficiency can be surmounted by a fundamental wave enhancement added by the linear control on the unit cells L1 and L2.

SUPPLEMENTARY NOTE 3: LINEAR CONTROL VS NO CONTROL

To show the impact of linear controls (on L1 and L2) on the fundamental wave magnitude of the pressure P_{NL} in front of the unit cell NL, Supplementary Figure. 3 compares the control off case and the case of pure linear control.

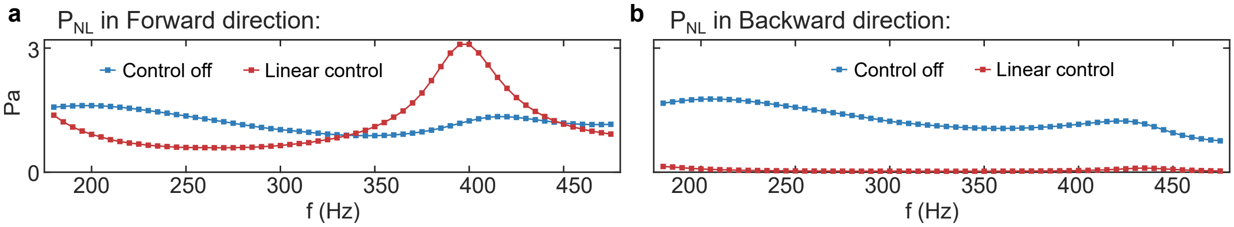

Supplementary Figure 3. **Effects of linear controls.** For the **a** forward and **b** backward configurations, the frequency responses of pressure P_{NL} in front of the unit NL derived from Fourier transform of time domain measurements, in the control off case and the case of pure linear control (applied on the unit cells L1 et L2), respectively.

One can identify from Supplementary Figure. 3 that in the forward configuration (Supplementary Figure. 3a), the applied linear controls enhance significantly the pressure P_{NL} in front of NL in the range of approximately $(370\,\mathrm{Hz}, 440\,\mathrm{Hz})$. This is why the higher harmonics are amplified in this range when the nonlinear control is imposed. On the contrary, for the range below 300 Hz, the linear control-induced reflection weakens the pressure P_{NL}. As a result, the harmonic conversion realized in this range (see Fig. 3 in the main manuscript) cannot be as good as in the range around 400 Hz, despite the improvement owing to the coincidence of a higher harmonic with the resonance. In the backward configuration (Supplementary Figure. 3b), as the pressure is almost eliminated by the linear controls, it is accordingly impossible to trigger nonlinear effects with the same nonlinear control laws as in the forward configuration.

SUPPLEMENTARY NOTE 4: HARMONIC DISTRIBUTIONS OF THE FULL CONTROL RESULTS

Here we provide more details about the achieved nonlinear nonreciprocal transmission. Supplementary Figure. 4a and Supplementary Figure. 4b display the harmonic distributions

(the first four harmonics) derived from the pressures measured and recorded over a duration of 10 s, under pure tone excitation at each of the considered frequencies (in a step of 5 Hz) in the range of [180 HZ, 475 HZ]. Both the pressure in front of the nonlinearly controlled unit cell (P_{NL}) and the final transmitted wave (P_{tr}) are illustrated, for the forward and backward configurations, respectively.

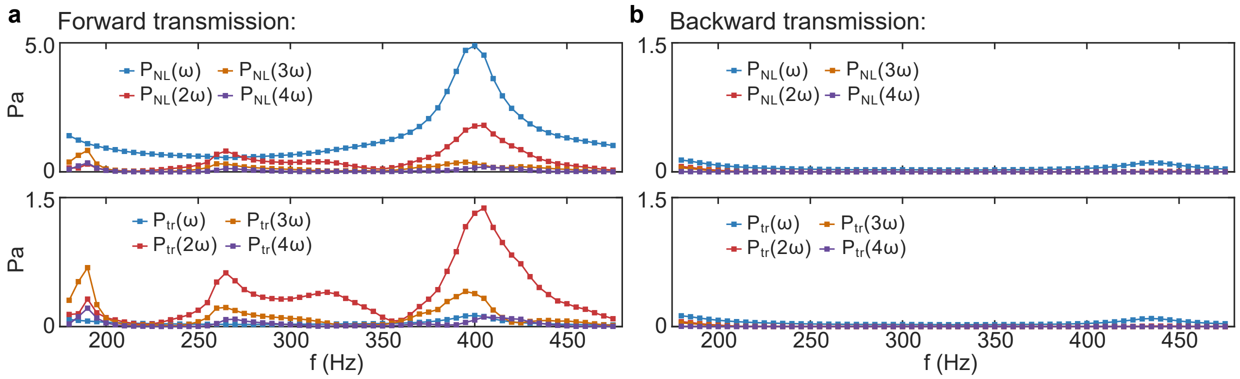

Supplementary Figure 4. **Nonreciprocal harmonic conversion under pure tone excitation.** **a**, **b** The first four harmonic components of the pressures in front of the unit cell subject to nonlinear control (P_{NL}) and of the final transmitted pressure (P_{tr}), in the forward and backward configurations, respectively.

In the range around 190 Hz, the final transmitted wave in the forward direction is dominated by the third harmonic, whereas in the range between 250 Hz and 330 Hz, the second harmonic is more pronounced. The manifestation of the higher harmonics is enabled in these ranges by matching with the resonance, as verified by the pure nonlinear control instances given in Supplementary Figure. 2c. However, the linear wave controls performed with L1 and L2 diminish the fundamental wave component of P_{NL} in these ranges, as we proved previously with Supplementary Figure. 3. In contrast, in the range around 400 Hz, where the resonance cannot facilitate the generation of higher harmonics, the final transmitted wave is largely amplified due to the fundamental wave enhancement offered by the linear controls, as confirmed previously with Supplementary Figure. 3a and also here by the response of P_{NL} in Supplementary Figure. 4a. The results for the backward configuration follow the prediction of the pure linear control case in Supplementary Figure. 3b, as nonlinearity cannot be excited when the fundamental component is mostly reflected.

SUPPLEMENTARY NOTE 5: NONRECIPROCAL TRANSMISSION ACHIEVED UNDER WAVE-PACKET EXCITATION

In the main manuscript, the preliminary assessment of the designed nonreciprocal converter has been carried out with pure tone excitation. Here we complete the study with additional measurements with wave-packet excitation. It is defined as a sine wave with Gaussian-type modulation: $p_{\mathrm{input}}(t) = p_0 \sin(2\pi f_{\mathrm{c}} t) \exp\left(-\frac{(t-t_0)^2}{\sigma}\right)$, where f_{c} denotes the center frequency of the pulse which lasts for 1 s, the variance σ is set to be 0.005 to provide a frequency bandwidth of around 20 Hz.

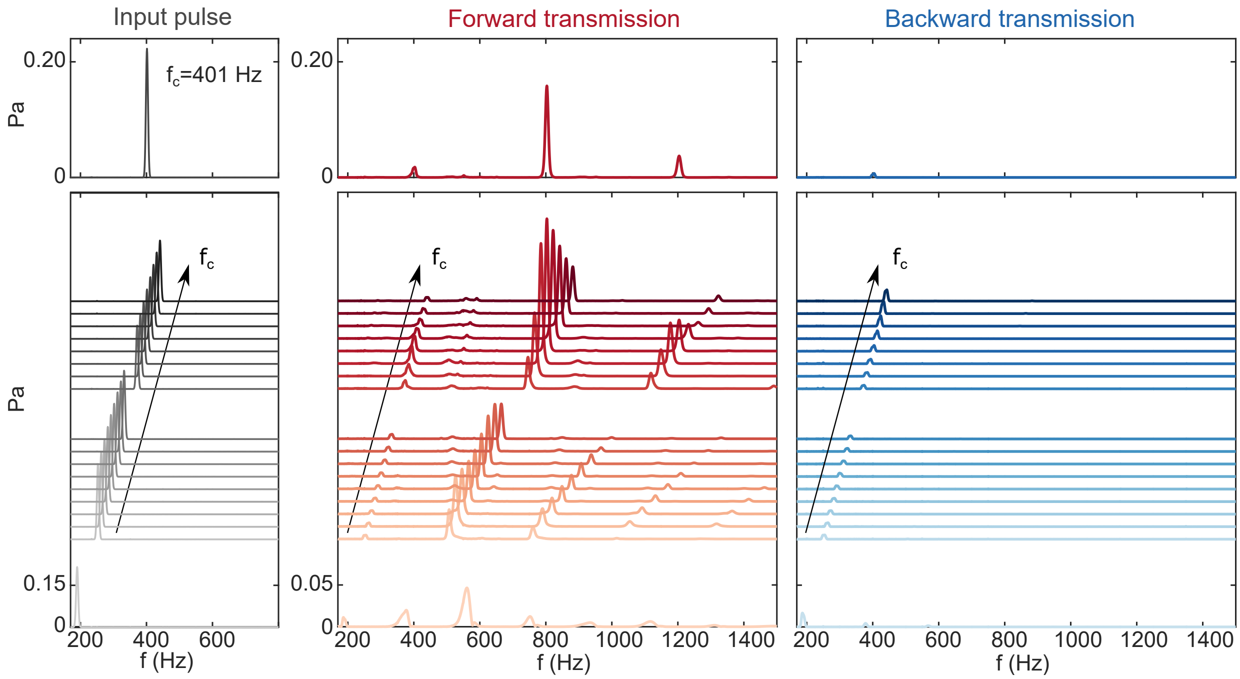

Supplementary Figure 5. **Nonreciprocal harmonic conversion under wave-packet excitation.** A Gaussian type modulation is accounted for to define different pulse excitation with a bandwidth of around 20 Hz and centered at frequency f_{c}. The input and the 2 transmitted spectra (in the forward and backward configurations, respectively) are depicted first for $f_{\mathrm{c}} \approx 401$ Hz (top), then for f_{c} varying in the effective nonreciprocal regions highlighted by the orange blocks in Fig. 3 in the main manuscript,

To handle such wave-packet excitation, the linear control bandwidth is enlarged based on the control laws used for Supplementary Figure. 4 (which makes the center frequency slightly shifted, for example from 405 Hz to 401 Hz in the top Figure of Supplementary

Figure. 5). The nonlinear control parameter $G_{\rm NL}$ is specified in the same way as the pure tone cases that the nonlinearity is strengthened to the greatest extent, provided that the whole system remains stable. A voltage level of 50 mVrms (higher than the previous case of sine excitation, but still remains very low) is imposed for all the present cases, corresponding to the input pressure in the range (2 Pa, 3 Pa). A period of 14 s is chosen for the time domain measurements and recording, during which the defined modulated sine source is repeated ten times. The final spectra illustrated in Supplementary Figure. 5 are obtained after averaging the Fourier transforms of all 10-time responses to the repeated inputs.

It is demonstrated that the achieved nonreciprocal conversion can still be very significant with multiple-frequency excitation. In the case of $f_{\rm c} = 401\,{\rm Hz}$ shown in the first row of Supplementary Figure. 5, we achieved a maximum spectrum magnitude of transmitted wave of 0.16 Pa in the forward configuration versus 0.007 Pa in the backward configuration. For ease of assessment, the definition of isolation ratio used by Popa et al.[2] is considered herein, namely $10\log_{10}(\int p_{\rm for}^2(t)dt/\int p_{\rm back}^2(t)dt)$ with $p_{\rm for}(t)$ and $p_{\rm back}(t)$ the transmitted pressure measured in the time domain for the forward and backward configurations, respectively. For the case of $f_{\rm c} = 401\,{\rm Hz}$, the derived results show an isolation ratio of 28 dB, together with only a tiny difference of 0.9 dB between the forward transmission and the input pressure.

Next, the same type of pulse excitation with different center frequencies $f_{\rm c}$ within the effective nonreciprocal transmission region (indicated by the orange blocks in Fig. 3 in the main manuscript) is taken into account in the second row of Supplementary Figure. 5. For the first effective narrow-band region near 190 Hz, an isolation ratio of 13.4 dB is confirmed. Regarding the second effective region from 250 Hz to 330 Hz, it is determined in the range of [17 dB, 23 dB]. The forward transmission in these two ranges is lower than the input pressure with a gap of 7 dB for the first and of around 10 dB for the second. The optimal nonreciprocal transmission occurs in the range of (380 Hz, 430 Hz) with the best at around 400 Hz, as presented by $f_c = 401\,{\rm Hz}$ described previously. In a nutshell, the pulse excitation results follow the trend of the sinusoidal excitation configuration, the nonreciprocity of the designed harmonic converter proves to be significant in the main range of (180 Hz, 475 Hz), which, offer us the opportunity to play unprecedentedly with real sound as finally achieved in the main manuscript.

SUPPLEMENTARY REFERENCES

[1] E. Rivet, *Modal Equalisation with Electroacoustic Absorbers*, Ph.D. thesis, Ecole Polytechnique Fédérale de Lausanne (2016).

[2] B.-I. Popa and S. A. Cummer, "Non-reciprocal and highly nonlinear active acoustic metamaterials," Nat. Commun. **5**, 3398 (2014).
