# Supplementary material for: Observation of non-reciprocal harmonic conversion in real sounds
Source: Commun Phys. 2023 May 6;6(1):93. doi: 10.1038/s42005-023-01217-w (PMC11041789; doi:10.1038/s42005-023-01217-w)
Supplement: Supplementary file 3 — Description of Additional Supplementary Files [file 42005_2023_1217_MOESM3_ESM.pdf]

Description of Additional Supplementary Files

File name: Supplementary Audio 1

Description: This audio file is a recording of the time-domain experimental results when using the note of the ocarina as the input. You will hear first the input note, then the forward and the backward transmitted notes. All signals were recorded directly in the experiments, without any post-processing.

File name: Supplementary Audio 2

Description: This audio file is a recording of the time-domain experimental results when using the note of the clarinet as the input. You will hear first the input note, then the forward and the backward transmitted notes. All signals were recorded directly in the experiments, without any post-processing.

File name: Supplementary Audio 3

Description: This audio file is a recording of the time-domain experimental results when using the note of the flute as the input. You will hear first the input note, then the forward and the backward transmitted notes. All signals were recorded directly in the experiments, without any post-processing.

File name: Supplementary Audio 4

Description: This audio file is a recording of the time-domain experimental results when using the note of the piano as the input. You will hear first the input note, then the forward and the backward transmitted notes. All signals were recorded directly in the experiments, without any post-processing.
